# A new approach to model the counts of earthquakes: INARPQX(1) process

**DOI:** 10.1007/s42452-020-04109-8

**Published:** 2021-02-03

**Authors:** Emrah Altun, Deepesh Bhati, Naushad Mamode Khan

**Affiliations:** 1 Department of Mathematics, Bartin University, 74100 Bartin, Turkey; 2 Department of Statistics, Central University of Rajasthan, Ajmer, India; 3 Department of Economics and Statistics, University of Mauritius, Reduit, Mauritius

**Keywords:** Discrete distribution, Earthquake, Over-dispersion, INAR(1) process, 62E15

## Abstract

This paper introduces a first-order integer-valued autoregressive process with a new innovation distribution, shortly INARPQX(1) process. A new innovation distribution is obtained by mixing Poisson distribution with quasi-xgamma distribution. The statistical properties and estimation procedure of a new distribution are studied in detail. The parameter estimation of INARPQX(1) process is discussed with two estimation methods: conditional maximum likelihood and Yule-Walker. The proposed INARPQX(1) process is applied to time series of the monthly counts of earthquakes. The empirical results show that INARPQX(1) process is an important process to model over-dispersed time series of counts and can be used to predict the number of earthquakes with a magnitude greater than four.

## Introduction

Destructive earthquakes are one of the biggest problems of all humanity. Earthquakes are not only natural disasters that threaten the people’s lives, but also affect the economies of the countries negatively. Therefore, statistical modeling of the counts of earthquakes and taking measures according to the model results are of great importance. Many different statistical models have been used in the literature to model earthquake events. Turkan and Ozel [26] used the linear regression, beta regression, and semi-parametric additive regression to model the casualty rate of earthquakes and related covariates. The relation between the magnitude of an earthquake and the number of deaths was investigated in several piece of researches such as Samardjieva and Badal [22], Gutiérrez et al. [10], Wyss [30], Koshimura et al. [15], Zhao et al. [31], and Wu et al. [29]. Besides these regression models, stochastic models are also powerful tools to model earthquakes. Stochastic point process models for earthquakes were discussed by Brillinger [7], Vere-Jones [27], Ogata [19, 20] and Zhuang et al. [32, 33]. Gospodinov and Rotondi [9] and Aktas et al. [1] used the compound Poisson process to model the cumulative energy release of mainshocks and the expected value of the earthquake, respectively. Ozel [21] introduced a bivariate compound Poisson model to predict the number of foreshocks and aftershocks.

The first-order integer-valued autoregressive process, shortly INAR(1), is an alternative model to predict the number of the time of series of counts such as monthly deaths from corona-virus, monthly counts of passengers for a specific airline company or monthly counts of destructive earthquakes for a specific region or a country. The INAR(1) process with Poisson innovations, known as INARP(1), was developed by McKenzie [17, 18] and Al-Osh and Alzaid [2] and increased its popularity in the last decade. The time series of counts generally display over-dispersion. The INARP(1) process can be used to model equi-dispersed time series of counts which means that the ratio of sample variance to sample mean should be one. The over-dispersion occurs when the sample variance is greater than the sample mean, the opposite indicates the under-dispersion. After the important researches of McKenzie [17, 18] and Al-Osh and Alzaid [2], the researches have focussed on the distribution of an innovation process of INAR(1) to develop new models for over-dispersed or under-dispersed time series of counts. In what follows, we list some recent contributions on overdispersed INAR(1) processes: INAR(1) process with geometric innovations (INARG(1)) by Jazi et al. [13], INAR(1) with Poisson–Lindley innovations (INARPL(1)) by Lívio et al. [16], compound Poisson INAR(1) by Schweer and Weiß [23], processes INAR(1) process with Katz family innovations by Kim and Lee [14], INAR(1) process with generalized Poisson and double Poisson innovations by Bourguignon et al. [6] , INAR(1) process with geometric marginals by Borges et al. [5] and INAR(1) process with Skellam innovations by Andersson and Karlis [4], INAR(1) process with a new Poisson-weighted exponential innovations by Altun [3].

In this study, we first introduce a new two-parameter discrete distribution as an alternative distribution for the case of over-dispersion. The proposed distribution is obtained by mixing the Poisson distribution with a quasi-xgamma distribution of Sen and Chandra [24]. The resulting distribution is called as Poisson-quasi-xgamma (PQX) distribution. The statistical properties of the PQX distribution are studied in detail as well as its parameter estimation with different methods of estimations. Then, we introduce a new INAR(1) model by using the PQX distribution as an innovation process and called this process as a INARPQX(1). The goal of the presented study is to open a new opportunity to model over-dispersed time series of counts with a more flexible innovation distribution than existing ones. To demonstrate the effectiveness of the proposed process, we use the earthquake data set (magnitude 4 and above) of Turkey. The earthquake data set is regularized as a monthly basis to predict the monthly counts of the earthquakes with magnitude 4 and above occurred in Turkey. We model the monthly counts of earthquakes by INAR(1) processes with several innovation distributions as well as PQX distribution.

The ongoing sections of the study are organized as follows. In Sect. 2, the PQX distribution is presented with its statistical properties. In Sect. 3, we discuss the parameter estimation procedure of the PQX distribution with maximum likelihood and method of moments estimation methods. In Sect. 4, Expectation–Maximization (EM) algorithm for the PQX distribution is given. Section 5 deals with the simulation study to compare the finite sample performance of the estimation methods. In Sect. 6, we introduce a new INAR(1) process with PQX innovations. Section 7 contains an application to the earthquake data of Turkey. We summarize the findings of the study in Sect. 8.

## Poisson-quasi-xgamma distribution

Let the random variable (rv) *X* follows a Poisson distribution. The probability mass function (pmf) of *X* is

1 $$\begin{aligned} \Pr \left( {X = x} \right) = \frac{{{\mathrm{{e}}^{ - \lambda }}{\lambda ^x}}}{{x!}},x = 0,1,2,..., \end{aligned}$$

where $\lambda >0$. The Poisson distribution is an attractive distribution and is widely used in many fields because of its tractable properties and software support. However, many count data sets exhibit over-dispersion or under-dispersion in which case the Poisson distribution does not work well. When the empirical variance is greater than the empirical mean, the over-dispersion occurs, and opposite case represents the under-dispersion. The negative-binomial (NB) distribution is a common choice for over-dispersed count data sets. However, in the last decade, researchers have introduced more sophisticated discrete probability distributions to open a new opportunity to model fat-tailed over-dispersed count data sets. Here, we introduce an alternative distribution to model over-dispersed time series of counts. To do this, the quasi-xgamma distribution, introduced by Sen and Chandra [24], is used as a mixing distribution for Poisson parameter $\lambda$. The probability density function (pdf) of quasi-xgamma distribution is

2 $$\begin{aligned} f\left( {x;\alpha ,\theta } \right) = \frac{\theta }{{1 + \alpha }}\left( {\alpha + \frac{{{\theta ^2}}}{2}{x^2}} \right) {\mathrm{{e}}^{ - \theta x}},x > 0 \end{aligned}$$

where $\alpha >0$ and $\theta >0$. The quasi-xgamma distribution is a special mixture of exponential and gamma densities and contains $\text {Gamma}\left( \theta ,3\right)$ (for $\alpha =0$) and $\text {xgamma}\left( \theta \right)$ (for $\alpha =\theta$), introduced by Sen et al. [25], distributions as its submodels. Now, we introduce a new two-parameter discrete distribution by mixing Poisson distribution with quasi-xgamma distribution.

### Proposition 1

Assume that the parameter $\lambda$ of the Poisson distribution follows a quasi-xgamma distribution. Then, the resulting distribution is

3 $$\begin{aligned}&\Pr \left( {X = x} \right) = \frac{{2\alpha \theta {{\left( {\theta + 1} \right) }^2} + {\theta ^3}\left( {x + 1} \right) \left( {x + 2} \right) }}{{2\left( {\alpha + 1} \right) {{\left( {\theta + 1} \right) }^{x + 3}}}},\nonumber \\&\quad x = 0,1,2,..., \end{aligned}$$

where $\alpha >0$ and $\theta >0$. Hereafter, we refer to (3) as $\text {PQX}\left( \alpha ,\theta \right)$.

### Proof

The proof is straightforward. $\square$

### Remark 1

The PQX distribution contains the below distributions as its sub-models or limiting case.

1. The PQX distribution reduces to Poisson-xgamma distribution for $\alpha =\theta$,
2. The PQX distribution reduces to $\text {NB}\left( 3,\theta / (\theta +1)\right)$ for $\alpha =0$,
3. The PQX distribution reduces to $\text {Geometric}\left( \theta / (\theta +1)\right)$ for $\alpha \rightarrow \infty$.

The corresponding cumulative distribution function (cdf) to (3) is

4 $$\begin{aligned} F\left( x \right) = \frac{{2\alpha + {{\left( {\theta + 1} \right) }^{ - x - 3}}\left( { - 2\alpha {{\left( {\theta + 1} \right) }^2} - \theta \left( {x + 3} \right) \left( {\theta \left( {x + 2} \right) + 2} \right) - 2} \right) + 2}}{{2\left( {\alpha + 1} \right) }}. \end{aligned}$$

The rest of the section is devoted to the inference of the statistical properties of PQX distribution and its parameter estimation.

### Statistical properties

#### Proposition 2

Let the rv *X* follow a PQX distribution. The factorial moments of *X* are given by

5 $$\begin{aligned} {\mu _{\left[ r \right] }} = \frac{{\Gamma \left( {r + 3} \right) + 2\alpha \Gamma \left( {r + 1} \right) }}{{2{\theta ^r}\left( {\alpha + 1} \right) }}. \end{aligned}$$

#### Proof

The factorial moments of *X* can be obtained by using mixing formula, as follows

$$\begin{aligned} {\mu _{\left[ r \right] }}= & {} \int \limits _0^\infty {\frac{{\theta {\lambda ^r}}}{{1 + \alpha }}\left( {\alpha + \frac{{{\theta ^2}}}{2}{\lambda ^2}} \right) {\mathrm{{e}}^{ - \theta \lambda }}\mathrm{{d}}\lambda } \\= & {} \frac{\theta }{{1 + \alpha }}\int \limits _0^\infty {{\lambda ^r}\left( {\alpha + \frac{{{\theta ^2}}}{2}{\lambda ^2}} \right) \exp \left( { - \theta \lambda } \right) \mathrm{{d}}\lambda } \\= & {} \frac{\theta }{{1 + \alpha }}\left\{ \int \limits _0^\infty {\alpha {\lambda ^r}\exp \left( { - \theta \lambda } \right) \mathrm{{d}}\lambda } \right. \\&\left. + \frac{{{\theta ^2}}}{2}\int \limits _0^\infty {{\lambda ^{r + 2}}\exp \left( { - \theta \lambda } \right) \mathrm{{d}}\lambda } \right\} \\= & {} \frac{\theta }{{1 + \alpha }}\left\{ {\frac{{\alpha \Gamma \left( {r + 1} \right) }}{{{\theta ^{r + 1}}}} + \frac{{{\theta ^2}\Gamma \left( {r + 3} \right) }}{{2{\theta ^{r + 3}}}}} \right\} \\= & {} \frac{\theta }{{1 + \alpha }}\left\{ {\frac{{\alpha \Gamma \left( {r + 1} \right) }}{{{\theta ^{r + 1}}}} + \frac{{\Gamma \left( {r + 3} \right) }}{{2{\theta ^{r + 1}}}}} \right\} \\= & {} \frac{{\Gamma \left( {r + 3} \right) + 2\alpha \Gamma \left( {r + 1} \right) }}{{2{\theta ^r}\left( {\alpha + 1} \right) }} \end{aligned}$$

The proof is completed. $\square$

Using (5), the mean and variance of PQX distribution are given, respectively, by

6 $$\begin{aligned} {\mathrm{E}}\left( X \right)= & {} \frac{{2\left( {\alpha + 3} \right) }}{{\theta \left( {2\alpha + 2} \right) }}, \end{aligned}$$

7 $$\begin{aligned} {\mathrm{Var}}\left( X \right)= & {} \frac{{{\alpha ^2} + \left( {\alpha + 1} \right) \left( {\alpha + 3} \right) \theta + 8\alpha + 3}}{{{{\left( {\alpha + 1} \right) }^2}{\theta ^2}}}. \end{aligned}$$

The dispersion index (Var(X)/E(X)) of PQX is given by

8 $$\begin{aligned} {\mathrm{DI}} = 1 + \frac{{\alpha \left( {\alpha + 8} \right) + 3}}{{\left( {\alpha + 1} \right) \left( {\alpha + 3} \right) \theta }}. \end{aligned}$$

As seen from (8), since the parameter $\alpha$ and $\theta$ are greater than zero, the dispersion index of PQX distribution is always greater than 1 which ensures that the PQX distribution is over-dispersed.

#### Proposition 3

The probability generating function (pgf) of PQX distribution is given by

9 $$\begin{aligned} G\left( s \right) = \frac{\theta }{{1 + \alpha }}\left[ {\frac{{{\theta ^2}}}{{{{\left( {\theta - s + 1} \right) }^3}}} + \frac{\alpha }{{\left( {\theta - s + 1} \right) }}} \right] . \end{aligned}$$

#### Proof

The pgf of *X* can be written in the form

10 $$\begin{aligned} G\left( s \right) = \int \limits _0^\infty {\exp \left[ {\lambda \left( {s - 1} \right) } \right] f\left( {\lambda ;\beta \theta } \right) } \mathrm{{d}}\lambda . \end{aligned}$$

Using (10), the pgf of *X* can be obtained as follows

$$\begin{aligned} G\left( s \right)= & {} \int \limits _0^\infty {\exp \left[ {\lambda \left( {s - 1} \right) } \right] \left( {\alpha + \frac{{{\theta ^2}}}{2}{\lambda ^2}} \right) {\mathrm{{e}}^{ - \theta \lambda }}} \mathrm{{d}}\lambda \\= & {} \frac{\theta }{{1 + \alpha }}\int \limits _0^\infty {\left( {\alpha + \frac{{{\theta ^2}}}{2}{\lambda ^2}} \right) \exp \left[ {\lambda \left( {s - \theta - 1} \right) } \right] \mathrm{{d}}\lambda } \\= & {} \frac{\theta }{{1 + \alpha }}\left[ {\frac{{{\theta ^2}}}{{{{\left( {\theta - s + 1} \right) }^3}}} + \frac{\alpha }{{\left( {\theta - s + 1} \right) }}} \right] \end{aligned}$$

$$\square $$

The moment generating function of PQX distribution can be easily obtained by substituting *s* with $\exp \left( t\right)$ in (9). Then, we have

11 $$\begin{aligned} M\left( t \right) = \frac{\theta }{{1 + \alpha }}\left[ {\frac{{{\theta ^2}}}{{{{\left( {\theta - \exp \left( t \right) + 1} \right) }^3}}} + \frac{\alpha }{{\left( {\theta - \exp \left( t \right) + 1} \right) }}} \right] . \end{aligned}$$

The quasi-xgamma distribution is a special mixture of $\text {exponential}\left( \theta \right)$ and $\text {gamma}\left( 3,\theta \right)$ distributions with mixing proportion, $p=\alpha /\left( \alpha +1\right)$. This property of the quasi-xgamma distribution can be used to generate rvs from PQX distribution. The below algorithm is given based on the mixture property of the quasi-xgamma.
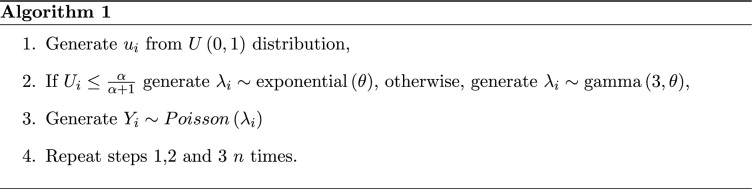


The skewness and kurtosis measures of PQX distribution can be easily obtained based on the first four raw moments by using

12 $$\begin{aligned}&S=\frac{\begin{array}{l} {\alpha }^3\, {\theta }^2 + 3\, {\alpha }^3\, \theta + 2\, {\alpha }^3 + 5\, {\alpha }^2\, {\theta }^2 + 27\, {\alpha }^2\, \theta \\ + 30\, {\alpha }^2 + 7\, \alpha \, {\theta }^2 + 33\, \alpha \, \theta + 18\, \alpha + 3\, {\theta }^2 + 9\, \theta + 6 \end{array}}{{\left( 8\, \alpha + 3\, \theta + 4\, \alpha \, \theta + {\alpha }^2\, \theta + {\alpha }^2 + 3\right) }^{\frac{3}{2}}}, \end{aligned}$$

13 $$\begin{aligned}&K=\frac{\begin{array}{ll} {\alpha }^4\, {\theta }^3 + 10\, {\alpha }^4\, {\theta }^2 + 18\, {\alpha }^4\, \theta + 9\, {\alpha }^4 + 6\, {\alpha }^3\, {\theta }^3 + 94\, {\alpha }^3\, {\theta }^2 \\ + 264\, {\alpha }^3\, \theta + 192\, {\alpha }^3 + 12\, {\alpha }^2\, {\theta }^3 + 206\, {\alpha }^2\, {\theta }^2 + 516\, {\alpha }^2\, \theta + 306\, {\alpha }^2 \\ + 10\, \alpha \, {\theta }^3 + 170\, \alpha \, {\theta }^2 + 360\, \alpha \, \theta + 216\, \alpha + 3\, {\theta }^3 + 48\, {\theta }^2 + 90\, \theta + 45 \end{array}}{{\left( 8\, \alpha + 3\, \theta + 4\, \alpha \, \theta + {\alpha }^2\, \theta + {\alpha }^2 + 3\right) }^2}. \end{aligned}$$

**Fig. 1:**
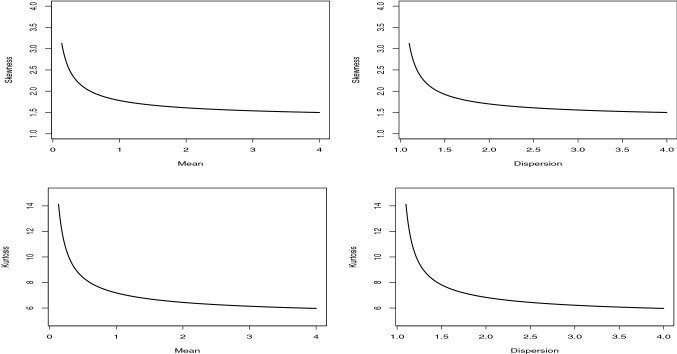
The skewness and kurtosis plots of the PQX distribution against the mean and dispersion values

The skewness and kurtosis plots of the PQX distribution against the mean and dispersion values are displayed in Fig. 1. The parameter $\alpha$ is taken 1 and parameter $\theta$ is increased in the interval (0.5, 15). From these figures, we conclude that the skewness and kurtosis decrease when the mean and dispersion increase.

The pmf of the NB distribution is

14 $$\begin{aligned} p\left( {y;\alpha ,p} \right) = \frac{{\Gamma \left( {y + \theta } \right) }}{{\Gamma \left( {y + 1} \right) \Gamma \left( \theta \right) }}{\left( {1 - p} \right) ^\theta }{p^y}, \end{aligned}$$

where $\theta >0$, $p \in (0,1)$. The mean, variance, skewness, and kurtosis values of the NB distribution are given, respectively, by

$$\begin{aligned} E\left( Y \right)= & {} \frac{{\theta p}}{{1 - p}} ,\\ \mathrm{{Var}}\left( Y \right)= & {} \frac{{\theta p}}{{{{\left( {1 - p} \right) }^2}}}, \\ S\left( Y \right)= & {} \frac{{1 + p}}{{\sqrt{\theta p} }}, \\ K\left( Y \right)= & {} \frac{6}{\theta } + \frac{{{{\left( {1 - p} \right) }^2}}}{{\theta p}}. \end{aligned}$$

**Fig. 2:**
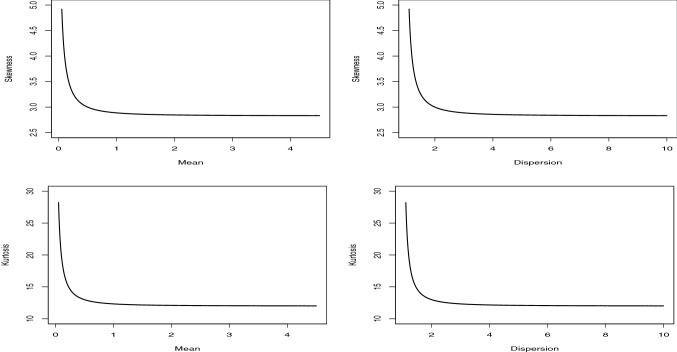
The skewness and kurtosis plots of the NB distribution against the mean and dispersion values

**Fig. 3:**
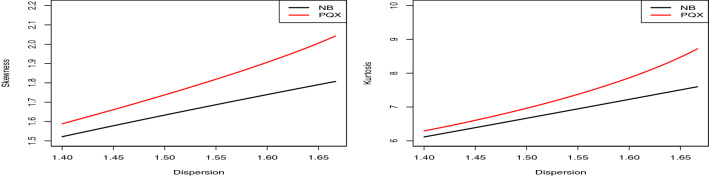
The skewness and kurtosis comparison of the mean-parametrized NB and PQX distributions

The skewness and kurtosis plots of the NB distribution against the mean and dispersion values are displayed in Fig. 2. The parameter $\theta$ is taken 0.5, and parameter *p* is increased in the interval (0.1, 0.9). From these figures, we conclude that the skewness and kurtosis decrease when the mean and dispersion increase. So, the NB and PQX distributions have the similar behaviors. However, to understand the differences between these distributions, we compare the mean-parametrized versions of both distributions. Substituting $p=\mu / (\mu +\theta )$ in (14), the mean-parametrized NB distribution is given by

15 $$\begin{aligned} p\left( {y;\alpha ,\mu } \right) = \frac{{\Gamma \left( {y + \theta } \right) }}{{\Gamma \left( \theta \right) \Gamma \left( {y + 1} \right) }}{\left( {\frac{\theta }{{\mu + \theta }}} \right) ^\theta }{\left( {\frac{\mu }{{\mu + \theta }}} \right) ^y}, \end{aligned}$$

where $\theta >0$, $\mu >0$ and $E\left( Y\right) =\mu$. Substituting $\alpha = {{\left( {3 - \theta \mu } \right) } / {\left( {\theta \mu - 1} \right) }}$ in (3), we have the pmf of the mean-parametrized PQX, given by

16 $$\begin{aligned}&\Pr \left( {X = x} \right) = \frac{{2{{\left( {3 - \theta \mu } \right) } / {\left( {\theta \mu - 1} \right) }}\theta {{\left( {\theta + 1} \right) }^2} + {\theta ^3}\left( {x + 1} \right) \left( {x + 2} \right) }}{{2\left( {{{\left( {3 - \theta \mu } \right) } / {\left( {\theta \mu - 1} \right) }} + 1} \right) {{\left( {\theta + 1} \right) }^{x + 3}}}},\nonumber \\&\quad x = 0,1,2,..., \end{aligned}$$

where $\theta >0$, $\mu >0$, $1<\theta \mu <3$ and $E\left( X\right) =\mu$. Figure 3 displays the skewness and kurtosis values of the mean-parametrized NB and PQX distributions against their dispersion values. The mean parameter is determined as 1 for both distributions. The parameter $\theta$ and $\alpha$ are increased in the interval (1.5, 2.5) since we have condition on the parameter space of the mean-parametrized PQX distributions, such as $1<\theta \mu <3$. As seen from these figures, when the dispersion increases, the skewness and kurtosis increase for both distributions. However, under these re-parametrization, the PQX distribution is able to model wider range of skewness and kurtosis than the NB distribution.

We also compare the right-tail probabilities of the mean-parametrized NB and PQX distributions. Table 1 lists the tail probabilities of the NB and PQX distributions for some values of the mean parameter $\mu$ and $\theta$. The results given in Table 1 indicate that the PQX has fatter right-tail than the NB distribution.

**Table 1:** The comparison of tail probabilities of the NB and PQX distributions

Tail probabilities	Models	$\mu =1$			$\mu =0.5$		
		$\theta =1.5$	$\theta =2$	$\theta =2.5$	$\theta =3$	$\theta =4$	$\theta =5$
$Pr(X > 5)$	NB	0.00973	0.00686	0.00524	0.00018	0.00012	0.00009
	PQX	0.01552	0.01052	0.00666	0.00124	0.00065	0.00034
$Pr(X>10)$	NB	0.00013	$4.704\times 10^{-5}$	$2.117\times 10^{-5}$	$2.979\times 10^{-8}$	$8.406\times 10^{-9}$	$3.379\times 10^{-9}$
	PQX	0.00036	0.00011	$3.329\times 10^{-5}$	$2.943\times 10^{-6}$	$5.431\times 10^{-7}$	$1.164\times 10^{-7}$
$Pr(X>15)$	NB	$1.566\times 10^{-6}$	$2.710\times 10^{-7}$	$6.643\times 10^{-8}$	$3.449\times 10^{-12}$	$3.757\times 10^{-13}$	$7.394\times 10^{-14}$
	PQX	$6.717\times 10^{-6}$	$8.492\times 10^{-7}$	$1.215\times 10^{-7}$	$5.384\times 10^{-9}$	$3.337\times 10^{-10}$	$2.901\times 10^{-11}$
$Pr(X>20)$	NB	$1.819\times 10^{-8}$	$1.434\times 10^{-9}$	$1.838\times 10^{-10}$	$4.441\times 10^{-16}$	$2.220\times 10^{-16}$	$2.220\times 10^{-16}$
	PQX	$1.097\times 10^{-7}$	$5.672\times 10^{-9}$	$3.778\times 10^{-10}$	$8.508\times 10^{-12}$	$1.747\times 10^{-13}$	$6.106\times 10^{-15}$

### Shape of PQX$(\alpha ,\theta )$ distribution

Using (3), the ratio of successive probabilities $P(X=x+1)/P(X=x)$ for PQX$(\alpha ,\theta )$ is given as

17 $$\begin{aligned}&\frac{P(X=x+1)}{P(X=x)}=\frac{(2+x)(3+x)\theta ^2+2\alpha (1+\theta )^2}{(1+\theta )\left( (1+x)(2+x)\theta ^2+2\alpha (1+\theta )^2 \right) }, \nonumber \\&\quad x=0,1,2,\ldots \end{aligned}$$

Further, the ratio $\frac{P(X=x+1)}{P(X=x)} < (>)1$ implies that the pmf is decreasing (increasing). Hence, solving the equation $\frac{P(X=x+1)}{P(X=x)}=1$ for non-integer *x* (say), the roots are given as

18 $$\begin{aligned} x_0^*= & {} \frac{2-3\theta }{2\theta }-\frac{\theta +2}{2\theta }\sqrt{1-8\alpha \left( \frac{1+\theta }{2+\theta }\right) ^2} \quad \text {and} \nonumber \\ x_0^{**}= & {} \frac{2-3\theta }{2\theta }+\frac{\theta +2}{2\theta }\sqrt{1-8\alpha \left( \frac{1+\theta }{2+\theta }\right) ^2} \end{aligned}$$

Hence, it can be easily verified as that

i. If $\left( 0<\theta<\frac{2}{3} \cap \alpha> \frac{(2+\theta )^2}{8(1+\theta )^2}\right) \cup \left( \frac{2}{3}\le \theta <2 \cap \alpha> \frac{\theta (2-\theta )}{(1+\theta )^2}\right) \cup (\theta \ge 2 \cap \alpha >0 )$, the ratio is less than 1; hence, the PQX$(\alpha ,\theta )$ is unimodal with mode at 0.
ii. If $\frac{2}{3}\le \theta <2 \cap \alpha = \frac{\theta (2-\theta )}{(1+\theta )^2}$, then mode of PQX$(\alpha ,\theta )$ is at 1.
iii. If $$0<\theta<2 \cap 0<\alpha <\frac{\theta (2-\theta )}{(1+\theta )^2}$$, then the PQX$$(\alpha ,\theta )$$ is unimodal and the mode is at $$\lceil x_0^{**} \rceil $$, where $$\lceil . \rceil $$ denote the ceiling function.
iv. If $$\left( 0<\theta<\frac{2}{3} \cap \frac{\theta (2-\theta )}{(1+\theta )^2} <\alpha \le \frac{(2+\theta )^2}{8(1+\theta )^2}\right) $$, PQX$$(\alpha ,\theta )$$ is bimodal with the mode at 0 and $$\lceil x_0^{**} \rceil $$.

**Fig. 4:**
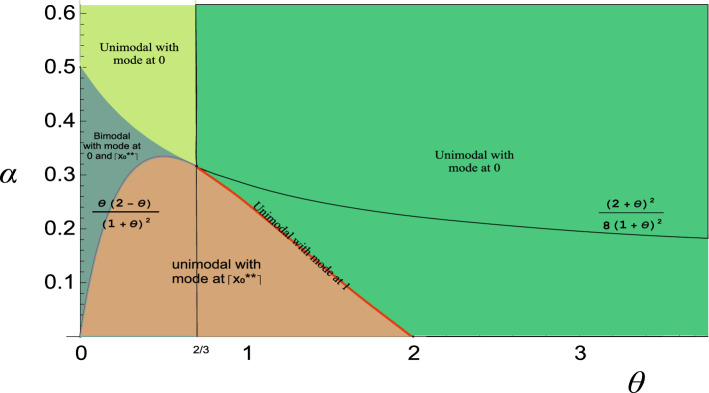
The modality of the PQX distribution

The results given above are graphically summarized in Fig. 4 which displays the modality regions of the PQX distribution with respect to the values of the parameters $\alpha$ and $\theta$.

**Fig. 5:**
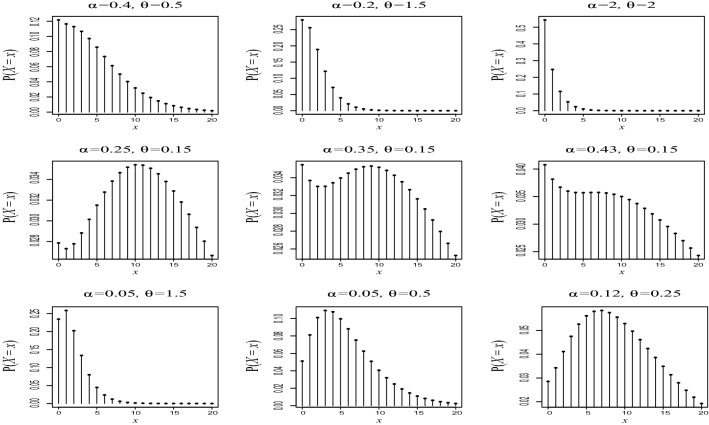
Probability mass function of ${PQX}(\alpha ,\theta )$ distribution for different combinations of the parameter values

In Fig. 5, we depict different shapes of PQX$(\alpha ,\theta )$ distribution for different parameters values. As seen from these figures, PQX distribution has bimodal and unimodal shapes with right skewness and symmetric. Moreover, for large value for $\alpha$ and $\theta$ the PQX distribution can be used to zero inflated count data sets.

## Estimation

### Maximum likelihood

Let $x_{1},x_{2},\dots ,x_{n}$ be a random observations from the $\text {PQX}$ distribution. The log-likelihood function is

19 $$\begin{aligned}&\ell \left( {\alpha ,\theta } \right) = \sum \limits _{i = 1}^n {\log \left( {2\alpha \theta {{\left( {\theta + 1} \right) }^2} + {\theta ^3}\left( {{x_i} + 1} \right) \left( {{x_i} + 2} \right) } \right) } \nonumber \\&\quad - n\log \left( {2\left( {\alpha + 1} \right) } \right) - \log \left( {\theta + 1} \right) \sum \limits _{i = 1}^n {\left( {{x_i} + 3} \right) } \end{aligned}$$

The partial derivatives of (19) with respect to $\alpha$ and $\theta$, the following score vectors are obtained.

20 $$\begin{aligned} \frac{{\partial \ell }}{{\partial \theta }}= & {} \sum \limits _{i = 1}^n {\frac{{4\alpha \left( {\theta + 1} \right) \theta + 2\alpha {{\left( {\theta + 1} \right) }^2} + 3{\theta ^2}\left( {{x_i} + 1} \right) \left( {{x_i} + 2} \right) }}{{2\alpha \theta {{\left( {\theta + 1} \right) }^2} + {\theta ^3}\left( {{x_i} + 1} \right) \left( {{x_i} + 2} \right) }}} \nonumber \\&+ \sum \limits _{i = 1}^n {\frac{{{x_i} + 3}}{{\theta + 1}}}, \end{aligned}$$

21 $$\begin{aligned} \frac{{\partial \ell }}{{\partial \alpha }}= & {} \sum \limits _{i = 1}^n {\frac{{2\theta {{\left( {\theta + 1} \right) }^2}}}{{2\alpha \theta {{\left( {\theta + 1} \right) }^2} + {\theta ^3}\left( {{x_i} + 1} \right) \left( {{x_i} + 2} \right) }}} - \frac{n}{{\alpha + 1}}. \end{aligned}$$

The simultaneous solution of (20) and (21) gives the maximum likelihood estimates (MLEs) of $\alpha$ and $\theta$. However, since the likelihood equations contain nonlinear functions, there is no explicit form for the MLEs of the parameters of PQX distribution. Therefore, the log-likelihood function in (19) should be maximized by using statistical software such as R, S-PLUS, Mathematica or MATLAB. Here, **nlm** function of R software is used to minimize the minus of log-likelihood function which is equivalent to maximization of log-likelihood. The inverse of observed information matrix evaluated at MLEs of the parameters is used to obtain corresponding standard errors. To do this, **fdHess** function of R software is used. The asymptotic confidence intervals of the parameters are obtained by

$$\begin{aligned} \widehat{\alpha }\pm z_{p/2}\sqrt{ {\mathrm{{Var}}({\widehat{\alpha }})}},\quad \widehat{\theta } \pm z_{p/2}\sqrt{{\mathrm{{Var}}(\widehat{\theta })}}, \end{aligned}$$

where $z_{p/2}$ is the upper *p*/2 quantile of the standard normal distribution.

### Method of moments

The method of moments estimators of the parameters $\alpha$ and $\theta$ can be obtained by equating theoretical moments of PQX distribution to sample moments, given as follows

22 $$\begin{aligned} {\mu '_1}= & {} \frac{{\left( {\alpha + 3} \right) }}{{\theta \left( {\alpha + 1} \right) }} = {m_1}, \end{aligned}$$

23 $$\begin{aligned} {\mu '_2}= & {} \frac{{\alpha \theta + 2\alpha + 3\theta + 12}}{{{\theta ^2}\left( {\alpha + 1} \right) }} = {m_2}, \end{aligned}$$

where $m_1$ and $m_2$ are the first and second sample moments, respectively. Simultaneous solution of (22) and (23) yields to following results

24 $$\begin{aligned} {\hat{\theta } _{MM}}= & {} \frac{{{{\hat{\alpha } }_{MM}} + 3}}{{{m_1}\left( {1 + {{\hat{\alpha } }_{MM}}} \right) }}, \end{aligned}$$

25 $$\begin{aligned} {\hat{\alpha } _{MM}}= & {} \frac{{ - 7m_1^2 + \sqrt{25m_1^4 + 12m_1^3 - 12m_1^2{m_2}} - 3\left( {{m_1} - {m_2}} \right) }}{{2m_1^2 + {m_1} - {m_2}}}. \end{aligned}$$

#### Proposition 4

For fixed $\alpha$, the MM estimator $\hat{\theta }$ of $\theta$ is positively biased.

#### Proof

Let ${\hat{\theta } _{MM}}=g(\bar{x})$ where $g\left( t \right) = {{\left( {\alpha + 3} \right) } / {\left( {t\left( {1 + \alpha } \right) } \right) }}$ for $t>0$. Taking the second partial derivative of *g*(*t*), we have

26 $$\begin{aligned} g''\left( t \right) = \frac{{2\left( {\alpha + 3} \right) }}{{\left( {\alpha + 1} \right) {t^3}}} > 0 \end{aligned}$$

which ensures that the *g*(*t*) is strictly convex. Using the Jensen’s inequality,

27 $$\begin{aligned} E\left( {g\left( {\bar{X}} \right) } \right) > g\left( {E\left( {\bar{X}} \right) } \right) \end{aligned}$$

So, since $g\left( {E\left( {\bar{X}} \right) }\right) =g(\mu )=g\left[ {{{2\left( {\alpha + 3} \right) } / {\left( {\theta \left( {2\alpha + 2} \right) } \right) }}} \right] =\theta$, we obtain $E\left( \hat{\theta }_{MM}\right) >\theta$. $\square$

#### Proposition 5

For fixed $\alpha$, the MM estimator $\hat{\theta }$ of $\theta$ is consistent and asymptotically normal28where

29 $$\begin{aligned} {v^2}\left( \theta \right) = \frac{{{\theta ^2}\left[ {\alpha \left( {8 + \alpha } \right) + \left( {\alpha + 1} \right) \left( {\alpha + 3} \right) \theta + 3} \right] }}{{{{\left( {\alpha + 3} \right) }^2}}}. \end{aligned}$$

#### Proof

Since $g'\left( \mu \right)$ exists and is nonzero valued, by the delta method, we have30where $g\left( {\bar{x}} \right) =\hat{\theta }$, $g\left( {\mu } \right) =\theta$ and

31 $$\begin{aligned} g'\left( \mu \right) = - \frac{{\left( {\alpha + 1} \right) {\theta ^2}}}{{\alpha + 3}} \end{aligned}$$

The proof is completed. $\square$

## EM algorithm for PQX$(\alpha ,\theta )$ distribution

In this section, we deals in the estimation of parameter $\theta$ and $\alpha$ of *PQX* distribution by another estimation method known as Expectation–Maximization (EM) (see Dempster et al., [8]). The EM algorithm consists of two steps: the E-step and the M-step. E-Step computes the expectation of the unobservable part given the current values of the parameters and M-step maximizes the complete data likelihood and updates the parameters using the conditional expectations obtained in E-step. This procedure can be useful when there are no closed-form expressions for estimating the parameters and the derivatives of the likelihood are complicated.

To start with, a hypothetical complete-data distribution is defined with joint probability function

32 $$\begin{aligned} g(X,\lambda ;\theta ,\alpha )= \frac{\theta \lambda ^x \mathrm{{e}}^ {-(1+\theta )\lambda }\left( 2\alpha +\theta ^2 \lambda ^2\right) }{2(1+\alpha ) x!}, \quad \quad \theta>0, \alpha >0. \end{aligned}$$

It is straightforward to verify that the E-step of an EM cycle requires the computation of the conditional expectations of $\left( \frac{\lambda ^2}{2\alpha +\lambda ^2\theta ^2} |x_i;\theta ^{(h)},\alpha ^{(h)}\right)$, $\left( \frac{1}{2\alpha +\lambda ^2\theta ^2} |x_i;\theta ^{(h)},\alpha ^{(h)}\right)$ and $\left( \lambda |x_i;\theta ^{(h)},\alpha ^{(h)} \right)$, say $t_i^{(h)}$, $s_i^{(h)}$ and $u_i^{(h)}$, respectively, where $\left( \theta ^{(h)},\alpha ^{(h)} \right)$ is the current value of $(\theta , \alpha )$. Using,

$$\begin{aligned}&g(\lambda |x;\theta ,\alpha )=\frac{\mathrm{{e}}^{-(1+\theta ) \lambda } (1+\theta )^{x+3} \lambda ^x \left( 2 \alpha +\theta ^2 \lambda ^2\right) }{x! \left( 2 \alpha (1+\theta )^2+\theta ^2 (x+1) (x+2)\right) } \\&t_i^{(h)}=\mathbb {E}\left( \frac{\lambda ^2}{2\alpha +\lambda ^2\theta ^2} \Big |x_i;\theta ^{(h)},\alpha ^{(h)}\right) \\&\quad =\frac{(x_i+1)(x_i+2)}{2\alpha ^{(h)} (1+\theta ^{(h)})^2+(\theta ^{(h)})^2 (x_i+1)(x_i+2)}, \\&s_i^{(h)} = \mathbb {E}\left( \frac{1}{2\alpha +\lambda ^2\theta ^2} \Big |x_i;\theta ^{(h)},\alpha ^{(h)}\right) \\&\quad =\frac{\left( 1+\theta _i^{(h)}\right) ^2}{2\alpha ^{(h)} \left( 1+\theta ^{(h)}\right) ^2+(\theta ^{(h)})^2 (x_i+1)(x_i+2)}, \\&\text {and} \\&u_i^{(h)} = \mathbb {E}\left( \lambda |x_i;\theta ^{(h)},\alpha ^{(h)}\right) \\&\quad =\frac{(x_i+1) \left( 2 \alpha ^{(h)} \left( 1+\theta ^{(h)}\right) ^2+\left( \theta ^{(h)}\right) ^2 (x_i+2) (x_i+3)\right) }{(1+\theta ^{(h)}) \left( 2\alpha ^{(h)}\left( 1+\theta ^{(h)}\right) ^2+\left( \theta ^{(h)}\right) ^2 (x_i+1) (x_i+2)\right) }. \end{aligned}$$

The EM cycle completes with the M-step, involving complete data maximum likelihood over $(\theta , \alpha )$, with the missing $\lambda$’s replaced by their conditional expectations

$$\begin{aligned} \begin{aligned} \theta ^{(h+1)}&=\frac{\bar{u}^{(h)}+\sqrt{(\bar{(u)}^{(h)})^2-8(\bar{t}^{(h)})^2}}{4 \bar{t}^{(h)}}, \\ \alpha ^{(h+1)}&= \frac{1}{2\bar{s}^{(h)}-1}. \end{aligned} \end{aligned}$$

## Simulation

The finite sample performance of MM and MLE methods is compared via simulation study. The below simulation procedure is implemented for this purpose.

1. Determine the sample size *n* and the parameter values of PQX distribution, $\varvec{\Theta }=\left( \alpha ,\theta \right)$,
2. Generate random observations from PQX distribution for given *n* and parameter vector,
3. Using the random sample in step 2, estimate $\varvec{\Theta }$ with MLE and MM methods,
4. Repeat the steps 2 and 3 based on the replication number, *N*,
5. Using the estimated parameter vector, $\hat{\varvec{\Theta }}$, and true parameter vector, $\varvec{\Theta }$, calculate the biases, mean relative estimates (MREs) and mean square errors (MSEs) by using,
  33 $$\begin{aligned} \mathrm{{Bias}}= & {} \sum \limits _{j = 1}^N {\frac{{{{ {\hat{\varvec{\theta }} }_{i,j}}} - { \varvec{\Theta }_i}}}{N}},\,\,\,\,\mathrm{{MRE}} = \sum \limits _{j = 1}^N {\frac{{{{{{{{\hat{\varvec{\Theta }} }}}_{i,j}}} / {{{\varvec{\Theta }_i }}}}}}{N}}, \nonumber \\ \mathrm{{MSE}}= & {} \sum \limits _{j = 1}^N {\frac{{{{\left( {{{{\hat{\varvec{\Theta }} }}_{i,j}} - {{\varvec{\Theta }_i }}} \right) }^2}}}{N}{\mathrm{}}},\,\,\,\ i=1,2. \end{aligned}$$

**Fig. 6:**
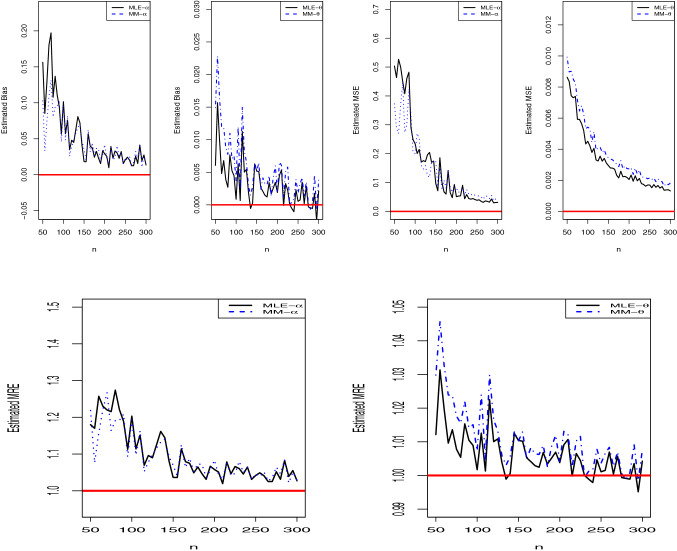
Estimated biases, MSEs, and MREs of the parameters of PQX distribution based on the MLE and MM estimation methods

The simulation is carried out with statistical software R. We generate $n=50,55,60,\ldots ,300$ sample of size from PQX distribution. The simulation replication number is $N=1000$. The true parameter vector is $\varvec{\Theta }=\left( \alpha =0.5,\theta =1.5\right)$. Figure 6 displays the simulation results. We expect that the estimated biases and MSEs should be near the zero for large *n* values. The results verify the expectation. The biases and MSEs approach the their nominal value, zero, for $n \rightarrow \infty$. Additionally, the MREs are near the one. The MLE and MM methods behave very similar for the parameter $\alpha$. However, the MLE method approaches the nominal values of biases, MSEs, and MREs more faster than MM method for the parameter $\theta$. Therefore, we suggest practitioners to use MLE method for estimating the parameters of PQX distribution.

## INAR(1) model with PQX innovations

Let the innovations $\left( \varepsilon _t\right) _{\mathbb {N}}$ be an independent and identically distributed (iid) process with ${\mathrm{E}}\left( {{\varepsilon _t}} \right) = {\mu _\varepsilon }$ and ${\mathrm{Var}}\left( {{\varepsilon _t}} \right) = \sigma _\varepsilon ^2$. The process, $\left( X_t\right) _{\mathbb {N}}$, is called as INAR(1) if it follows a recursion

34 $$\begin{aligned} {X_t} = p \circ {X_{t - 1}} + {\varepsilon _t}, \end{aligned}$$

where $0\le p <1$. The symbol, $\circ$, represents a binomial thinning operator and is defined as

35 $$\begin{aligned} p \circ {X_{t - 1}}: = \sum \limits _{j = 1}^{{X_{t - 1}}} {{Z_j}}, \end{aligned}$$

where $\left\{ Z _j \right\} _{j \ge 1}$ is referred to as a counting series and be a sequence of iid Bernoulli rvs with $\Pr \left( {{Z_j} = 1} \right) = p$. The INAR(1) process is stationary for $0\le p <1$. The process is non-stationary for the case $p=1$. The INAR(1) process is a homogeneous Markov chain with the one-step transition probabilities given by McKenzie [17], Al-Osh and Alzaid [2])

36 $$\begin{aligned}&\Pr \left( {\left. {{X_t} = k} \right| {X_{t - 1}} = l} \right) \nonumber \\&\quad =\sum \limits _{i = 1}^{\min \left( {k,l} \right) } {{\left( \begin{array}{l} l\\ i \end{array} \right) {p^i}{{\left( {1 - p} \right) }^{l - i}}}\Pr \left( {{\varepsilon _t} = k - i} \right) ,k,l \ge 0}. \end{aligned}$$

The mean, variance, and dispersion index of INAR(1) process are given, respectively, by Weiß [28]

37 $$\begin{aligned} \text {E}\left( {{X_t}} \right)= & {} \frac{{{\mu _\varepsilon }}}{{1 - p}}, \end{aligned}$$

38 $$\begin{aligned} \text {Var}\left( {{X_t}} \right)= & {} \frac{{p{\mu _\varepsilon } + \sigma _\varepsilon ^2}}{{1 - {p^2}}}, \end{aligned}$$

39 $$\begin{aligned} \text {DI}{_X}= & {} \frac{{\text {DI}_{\varepsilon } + p}}{{1 + p}}, \end{aligned}$$

where $\mu _\varepsilon$, $\sigma _\varepsilon ^2$ and $DI_{\varepsilon }$ are the mean, variance, and dispersion index of innovation distribution, respectively. In real-life problems, the empirical dispersion is greater than one (over-dispersion) in general. For instance, monthly counts of passengers, yearly number of destructive earthquakes, yearly number of goals of a and among others. To model these kind of datasets, the innovation distribution of INAR(1) process should be able to model over-dispersion. Here, we propose a new INAR(1) process with PQX innovations to model such data sets. Let ${\left\{ {{\varepsilon _t}} \right\} _{\mathbb {N}}}$ follows a PQX distribution given in (3). The one-step transition probability of INARPQX(1) model is given by

40 $$\begin{aligned}&\Pr \left( {\left. {{X_t} = k} \right| {X_{t - 1}} = l} \right) = \sum \limits _{i = 1}^{\min \left( {k,l} \right) } {\left( \begin{array}{l} l\\ i \end{array} \right) {p^i}{{\left( {1 - p} \right) }^{l - i}}} \nonumber \\&\quad \frac{{2\alpha \theta {{\left( {\theta + 1} \right) }^2} + {\theta ^3}\left( {k - i + 1} \right) \left( {k - i + 2} \right) }}{{2\left( {\alpha + 1} \right) {{\left( {\theta + 1} \right) }^{k - i + 3}}}} \end{aligned}$$

Frow now on, this process is called as INARPQX(1) process. Using the properties of INAR(1) process, the mean, variance, and dispersion index of INARPQX(1) process are given, respectively, by

41 $$\begin{aligned} {\mu _X}= & {} \frac{{\left( {\alpha + 3} \right) }}{{\theta \left( {\alpha + 1} \right) \left( {1 - p} \right) }}, \end{aligned}$$

42 $$\begin{aligned} \sigma _X^2= & {} \frac{{{\alpha ^2}\left( {\theta + \theta p + 1} \right) + 4\alpha \left( {\theta + \theta p + 2} \right) + 3\left( {\theta + \theta p + 1} \right) }}{{{\theta ^2}{{\left( {\alpha + 1} \right) }^2}\left( {1 - {p^2}} \right) }}, \end{aligned}$$

43 $$\begin{aligned} {\mathrm{D}}{{\mathrm{I}}_X}= & {} 1 + \frac{{\alpha \left( {\alpha + 8} \right) + 3}}{{\theta \left( {\alpha + 1} \right) \left( {\alpha + 3} \right) \left( {1 + p} \right) }}. \end{aligned}$$

According to the results given in Al-Osh and Alzaid [2], the conditional expectation and variance of INARPQX(1) process are given, respectively, by

44 $$\begin{aligned} \text {E}\left( {\left. {{X_t}} \right| {X_{t - 1}}} \right)= & {} p{X_{t - 1}} + \frac{{2\left( {\alpha + 3} \right) }}{{\theta \left( {2\alpha + 2} \right) }} \end{aligned}$$

45 $$\begin{aligned} {\mathrm{Var}}\left( {\left. {{X_t}} \right| {X_{t - 1}}} \right)= & {} p\left( {1 - p} \right) {X_{t - 1}} \nonumber \\&+ \frac{{{\alpha ^2} + \left( {\alpha + 1} \right) \left( {\alpha + 3} \right) \theta + 8\alpha + 3}}{{{{\left( {\alpha + 1} \right) }^2}{\theta ^2}}} \end{aligned}$$

### Estimation

The estimation procedure of INAR(1) process is discussed with three estimation methods. These are conditional maximum likelihood (CML), Yule–Walker (YL), and conditional least squares (CLS). The finite sample performance of these estimation methods is compared via extensive simulation study. The rest of this section is devoted to theoretical background of the used estimation methods.

#### Conditional maximum likelihood

Let $X_1,X_2,...,X_T$ be a random sample from the stationary process, INARPQX(1). The conditional log-likelihood function of INARPQX(1) is

46 $$\begin{aligned} \ell \left( \Theta \right)= & {} \sum \limits _{t = 2}^T {\ln \left[ {\Pr \left( {\left. {{X_t} = k} \right| {X_{t - 1}} = l} \right) } \right] } \nonumber \\= & {} \sum \limits _{t = 2}^T \ln \left[ \sum \limits _{i = 1}^{\min \left( {{x_t},{x_{t - 1}}} \right) } {\left( \begin{array}{l} {x_{t - 1}}\\ i \end{array} \right) {p^i}{{\left( {1 - p} \right) }^{{x_{t - 1}} - i}}} \right. \nonumber \\&\quad \left. \frac{{2\alpha \theta {{\left( {\theta + 1} \right) }^2} + {\theta ^3}\left( {{x_t} - i + 1} \right) \left( {{x_t} - i + 2} \right) }}{{2\left( {\alpha + 1} \right) {{\left( {\theta + 1} \right) }^{{x_t} - i + 3}}}} \right] \end{aligned}$$

The CML estimators of $\left( p,\alpha ,\theta \right)$ can be obtained by direct maximization of log-likelihood function given in (46). The **nlm** function of **R** software is used to minimize the minus of log-likelihood function. The inverse of observed information matrix is used to obtain corresponding standard errors of the CML estimation of the INARPQX(1) process. The observed information matrix is obtained by **fdHess** function of **R** software.

#### Yule–Walker

The YW estimators of INARPQX(1) process are obtained by equating the sample moments to the theoretical moments of the process. Since the autocorrelation function (ACF) of INAR(1) process at lag *h* is ${\rho _x}\left( h \right) = {p^h}$, the YW estimator of *p* is given by

47 $$\begin{aligned} {\hat{p}_{YW}} = \frac{{\sum \limits _{t = 2}^T {\left( {{X_t} - \bar{X}} \right) \left( {{X_{t - 1}} - \bar{X}} \right) } }}{{\sum \limits _{t = 1}^T {{{\left( {{X_t} - \bar{X}} \right) }^2}} }}. \end{aligned}$$

The YW estimators of $\alpha$ and $\theta$ are obtained by equating the sample mean and sample dispersion to theoretical mean and theoretical dispersion of the process. The YW estimator of $\theta$ is given by

48 $$\begin{aligned} {\hat{\theta } _{YW}} = \frac{{{{\hat{\alpha } }_{YW}} + 3}}{{\bar{X}\left( {1 + {{\hat{\alpha } }_{YW}}} \right) \left( {1 - {{\hat{p}}_{YW}}} \right) }}, \end{aligned}$$

where $\bar{X} = {{\sum \nolimits _{t = 1}^T {{X_t}} } / N}$. Substituting $\theta$ with (48) in (43) and equating (43) to sample dispersion, ${\widehat{DI}}_X$, we have

49 $$\begin{aligned} {\hat{\alpha } _{YW}} = \frac{{\left( \begin{array}{l} - 3{\widehat{\text {DI}}_X} + 4\bar{X} + {{\hat{p}}_{YW}}\left( { - 3{{\widehat{\text {DI}}}_X} - 4\bar{X} + 3} \right) \\ - \sqrt{\bar{X}\left( {{{\hat{p}}_{YW}} - 1} \right) \left( {12{{\widehat{\text {DI}}}_X} - 13\bar{X} + {{\hat{p}}_{YW}}\left( {12{{\widehat{\text {DI}}}_X} + 13\bar{X} - 12} \right) - 12} \right) } + 3 \end{array} \right) }}{{{{\widehat{\text {DI}}}_X} - \bar{X} + {{\hat{p}}_{YW}}\left( {{{\widehat{\text {DI}}}_X} + \bar{X} - 1} \right) - 1}}. \end{aligned}$$

### Model accuracy

The standardized Pearson residual is used to check the fitted model accuracy. The standardized Pearson residuals are given by

50 $$\begin{aligned} {e_t} = \frac{{{x_t} - {\mathrm{E}}\left( {\left. {{X_t}} \right| {x_{t - 1}}} \right) }}{{\sqrt{{\mathrm{Var}}\left( {\left. {{X_t}} \right| {x_{t - 1}}} \right) } }},{\mathrm{}}t = 2,3,...,T, \end{aligned}$$

where ${\mathrm{E}}\left( {\left. {{X_t}} \right| {x_{t - 1}}} \right)$ and ${\mathrm{Var}}\left( {\left. {{X_t}} \right| {x_{t - 1}}} \right)$ are given in (44) anf (45), respectively. When the model is adequate for fitted data set, the standardized Pearson residuals should be uncorrelated with zero mean and unit variance. If the variance of standardized Pearson residuals is higher/lower than one, it shows that there is more or less dispersion considered by fitted model (see, Harvey and Fernandes, [11]).

### Simulation

We compare the finite sample performance of CML and YW estimators of the parameters of INARPQX(1) process with a brief simulation study. We generate $n=100, 300$ and 500 sample of sizes from INAR(1) process with PQX innovations. The simulation replication number is $N=1,000$. The two parameter vectors are used: $\left( p=0.3,\alpha =0.5,\theta =0.5\right)$, $\left( p=0.3,\alpha =0.5,\theta =2\right)$. The simulation is carried out with **R** software. The results are interpreted based on the estimated biases, MSEs and MREs. The required formulas of these measures are given in Sect. 4. We expect to see that the estimated biases and MSEs approach to zero for large values of *n*. Beside this, the estimated MREs should be near the one. The simulation results are given in Table 2. Based on the results given in this table, it is clear that the estimated biases and MSEs are very near the their desired value, zero. Moreover, all estimated MREs are near the one. However, the CML estimators approach to the nominal values of MSEs and MREs more faster than those of YW estimators. Therefore, we suggest practitioners to use CML estimation method to obtain the unknown parameters of INARPQX(1) process. Actually, the both estimation methods work well for large sample sizes. So, if the number of time series of counts are large enough, YW estimation method is also preferable.

**Table 2:** Simulation results of INARPQX(1) process for CML and YW estimation methods

Sample size		$p=0.3$		$\alpha =0.5$		$\theta =0.5$	
		CML	YW	CML	YW	CML	YW
100	Bias	$-$0.0170	$-$0.0294	0.2888	0.0304	$-$0.0027	$-$0.0090
	MSE	0.0033	0.0085	0.4373	0.4743	0.0067	0.0075
	MRE	0.9661	0.9412	1.0578	1.0608	0.9945	0.9819
300	Bias	$-$0.0070	$-$0.0118	0.0093	0.0636	0.0014	$-$0.0037
	MSE	0.0012	0.0029	0.0711	0.2281	0.0016	0.0020
	MRE	0.9861	0.9764	1.0187	1.1273	1.0028	0.9926
500	Bias	$-$0.0020	$-$0.0067	0.0128	0.0259	0.0016	0.0008
	MSE	0.0006	0.0015	0.0424	0.1243	0.0011	0.0014
	MRE	0.9960	0.9866	1.0256	1.0518	1.0032	1.0017

		$p=0.3$		$\alpha =0.5$		$\theta =2$	
		CML	YW	CML	YW	CML	YW
100	Bias	$-$0.0126	$-$0.0191	$-$0.0461	0.1417	0.1381	0.0375
	MSE	0.0061	0.0097	0.1575	0.7691	0.2210	0.1819
	MRE	0.9581	0.9364	0.9079	1.2834	1.0691	1.0188
300	Bias	$-$0.0063	$-$0.0079	$-$0.0195	0.0977	0.0563	0.0229
	MSE	0.0020	0.0034	0.1093	0.4875	0.0831	0.0926
	MRE	0.9789	0.9735	0.9611	1.1955	1.0282	1.0115
500	Bias	$-$0.0022	$-$0.0025	0.0005	0.0651	0.0357	0.0257
	MSE	0.0014	0.0021	0.0794	0.2553	0.0475	0.0634
	MRE	0.9928	0.9918	1.0009	1.1303	1.0179	1.0129

## Empirical study

In this section, we illustrate the importance of the INARPQX(1) by an application on the earthquake data of Turkey. Firstly, we describe the created earthquake catalog and its properties. In the second step, INAR(1) processes defined under different innovation distributions are used to model the monthly counts of the earthquake.

### Study area and data

**Fig. 7:**
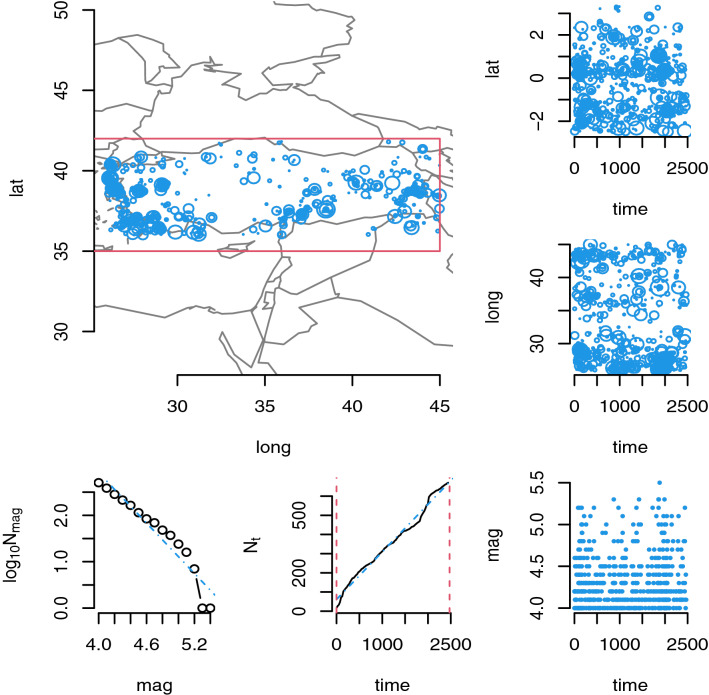
The plots of the earthquake catalog of Turkey

The earthquake data of the Turkey is obtained from the Disaster & Emergency Management Authority Presidential of Earthquake Department, Turkey. The data are available from https://deprem.afad.gov.tr/?lang=en. The used data contain the earthquakes with magnitude 4 and above occurred in Turkey between the dates 6 January 2012 and 14 October 2018. Firstly, the earthquake catalog of the Turkey is created by using the **ETAS** package of the **R** software. The detail on this package can be found in Jalilian [12]. The earthquake catalog is displayed in Fig. 7. The top-left figure shows the spatial distribution of the earthquakes under the study area. The three figures in the right part of Fig. 7 show the changes of the latitude, longitude, and magnitude of the earthquakes over the time. The two figures in the bottom-right part of Fig. 7 show the completeness and time stationary of the earthquake catalog. Here, $N_m$ represents the number of earthquakes with magnitude $\ge m$. If the plot of $\log (N_m)$ versus *m* shows linear trend, it represents the completeness of the earthquake catalog. Besides, the stationary of the earthquake catalog is evaluated based on the plot of $N_t$ versus *t*. Here, $N_t$ represents the number of earthquakes up to time *t*. If the plotted points of $N_t$ versus *t* have a functional form such as $N_t=\lambda _0 t$ where $\lambda >0$, it is an evidence that the time series of the earthquake is stationary. Therefore, we conclude that the used earthquake catalog is stationary and complete.

### Modeling of the number of earthquakes

The importance of INARPQX(1) model is demonstrated with an application to the monthly counts of earthquakes in Turkey. The proposed model, INARPQX(1), is compared with well-known INAR processes, INARP(1), INARPL(1), INARG(1), and INAR(1) with NB innovations shortly INARNB(1). The one-step translation probabilities of the used models are given, respectively, by

51 $$\begin{aligned}&\Pr \left( {\left. {{X_t} = k} \right| {X_{t - 1}} = l} \right) = \sum \limits _{i = 0}^{\min \left( {k,l} \right) } {\left( \begin{array}{l} l\\ i \end{array} \right) {p^i}{{\left( {1 - p} \right) }^{l - i}}} \nonumber \\&\quad \frac{{\exp \left( { - \lambda } \right) {\lambda ^{k - i}}}}{{\left( {k - i} \right) !}},\,\,\ \lambda > 0, \end{aligned}$$

52 $$\begin{aligned}&\Pr \left( {\left. {{X_t} = k} \right| {X_{t - 1}} = l} \right) = \sum \limits _{i = 0}^{\min \left( {k,l} \right) } {\left( \begin{array}{l} l\\ i \end{array} \right) {p^i}{{\left( {1 - p} \right) }^{l - i}}} \nonumber \\&\quad \frac{{{\theta ^2}\left( {k - i + \theta + 2} \right) }}{{{{\left( {\theta + 1} \right) }^{k - i + 3}}}},\,\,\ \theta > 0, \end{aligned}$$

53 $$\begin{aligned}&\Pr \left( {\left. {{X_t} = k} \right| {X_{t - 1}} = l} \right) = \sum \limits _{i = 0}^{\min \left( {k,l} \right) } {\left( \begin{array}{l} l\\ i \end{array} \right) {p^i}{{\left( {1 - p} \right) }^{l - i}}} \theta \nonumber \\&\quad \left( {1 - \theta } \right) ^{k - i},{\mathrm{}}\,\,\ 0< \theta < 1, \end{aligned}$$

54 $$\begin{aligned}&\Pr \left( {\left. {{X_t} = k} \right| {X_{t - 1}} = l} \right) = \sum \limits _{i = 0}^{\min \left( {k,l} \right) } {\left( \begin{array}{l} l\\ i \end{array} \right) {p^i}{{\left( {1 - p} \right) }^{l - i}}} \nonumber \\&\quad \left( {\begin{array}{c} {n + k - i - 1}\\ {k - i} \end{array}} \right) {\left( {1 - \pi } \right) ^{k - i}}{\pi ^n},{\mathrm{}} {\mathrm{}}n > 0,{\mathrm{}}0< \pi < 1. \end{aligned}$$

To analyze the reported data set in the previous section, the earthquakes are grouped monthly to predict the monthly number of the earthquakes with magnitude $\ge 4$. We use the $-\ell$, Akaike Information Criteria (AIC), and Bayesian Information Criteria (BIC) statistics to select the most appropriate model for the used data set. The lowest values of these criteria show the best fitted model. The used data sets consist of 82 monthly counts of earthquakes with magnitude greater than 4 between the date of January 2012 and March 2018. Firstly, the possible over-dispersion in the data set should be explored. For this aim, we use the hypothesis test for over-dispersion proposed by Schweer and Weiß [23]. The test statistic value of the over-dispersion hypothesis is 24.187, and its corresponding *p* value is $<0.001$ which indicates that the used data set displays over-dispersion. Therefore, the innovation distribution of the INAR(1) process should be able to capture the over-dispersion in the data set.

**Fig. 8:**
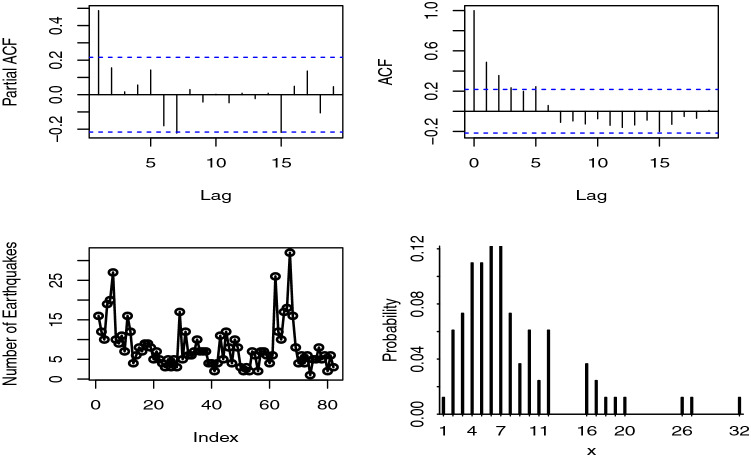
The plots of time series, autocorrelation, and partial autocorrelation functions for the monthly counts of earthquakes in Turkey

The some useful plots of the used data set displayed in Fig. 8. As seen from autocorrelation function (ACF) and partial ACF (PACF) plots, there is a clear cut of at first lag which ensures that the AR(1) process can be used to model this data set.

**Table 3:** The estimated parameters of the fitted INAR(1) models and corresponding goodness-of-fit statistics

Model	Parameters	Estimate	SE	$-\ell$	AIC	BIC	$\mu _X$	$\sigma ^2_X$	$\mathrm{{DI}}_X$
INARPQX(1)	*p*	0.461	0.042	221.300	448.599	455.819	7.948	31.723	3.991
	$\alpha$	94.964	4.342						
	$\theta$	0.238	0.076						
INARNB(1)	*p*	0.427	0.058	223.887	453.774	460.994	7.966	26.711	3.353
	$\phi$	0.229	0.053						
	n	1.358	0.460						
INARG(1)	*p*	0.460	0.041	224.299	452.599	457.412	7.948	42.084	5.295
	$\theta$	0.189	0.022						
INARPL(1)	*p*	0.418	0.046	224.377	452.755	457.568	7.969	24.755	3.106
	$\theta$	0.373	0.043						
INARP(1)	*p*	0.307	0.047	266.499	536.998	541.812	8.015	8.015	1
	$\lambda$	5.553	0.441						
Empirical							8.183	33.978	4.152

**Fig. 9:**
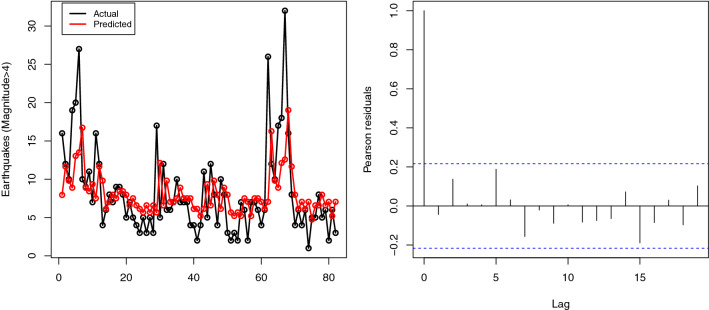
The predicted values of the monthly counts of earthquakes in Turkey

The estimated parameters of the fitted INAR(1) processes and corresponding AIC, BIC values are listed in Table 3. As seen from the results reported in Table 3, the proposed INARPQX(1) process has the lowest values of AIC and BIC values which indicates the proposed model performs better than INARP(1), INARG(1), INARPL(1), and IARNB(1) models. Moreover, we analyze the departure from error distribution by means of residual analysis. For this aim, we use the Pearson residuals. The Pearson residuals can be calculated by using

55 $$\begin{aligned} {r_t} = \frac{{{X_t} - E\left( {\left. {{X_t}} \right| {X_{t - 1}}} \right) }}{{\text {Var}{{\left( {\left. {{X_t}} \right| {X_{t - 1}}} \right) }^{{1 / 2}}}}} \end{aligned}$$

where $E\left( {\left. {{X_t}} \right| {X_{t - 1}}} \right)$ and $\text {Var}\left( {\left. {{X_t}} \right| {X_{t - 1}}} \right)$ are given in (44) and (45), respectively. When the fitted model is correct, the mean and variance of the Pearson residuals should be near the zero and one, respectively. Also, there should be no autocorrelation problem for the estimated Pearson residuals. The mean and variance of the Pearson residuals are calculated 0.0009 and 0.9754, respectively, which are very near the desired values. Additionally, the ACF plot of the Pearson residuals is displayed in right side of Fig. 9 which indicates that there is no autocorrelation problem for the Pearson residuals. The fitted INARPTE(1) model is given by

$$\begin{aligned} {X_t} = 0.461 \circ {X_{t - 1}} + {\varepsilon _t}. \end{aligned}$$

where ${\varepsilon _t} \sim \text {PQX}\left( 94.964,4.342\right)$. The predicted values of criminal mischief in 14th police car beat can be obtained by using (56).

56 $$\begin{aligned} {{\hat{X}}_1}= & {} \frac{{2\left( {\hat{\alpha } + 3} \right) }}{{2\hat{\theta } \left( {\hat{\alpha } + 1} \right) \left( {1 - \hat{p}} \right) }} = {\mathrm{7}}{\mathrm{.945}} \nonumber \\ {{\hat{X}}_t}= & {} \hat{p}{X_{t - 1}} + \frac{{2\left( {\hat{\alpha } + 3} \right) }}{{2\hat{\theta } \left( {\hat{\alpha } + 1} \right) }} = 0.432{X_{t - 1}} + {\mathrm{4}}{\mathrm{.287}} \end{aligned}$$

The predicted values of monthly counts of earthquakes are displayed in the left side of Fig. 9.

## Conclusion and future work

This study introduces a new two-parameter discrete distribution, shortly PQX, for modeling the over-dispersed counts. The statistical properties of the PQX distribution are derived and estimation of the unknown parameters of the proposed distribution is discussed in detail. INARPQX(1) processes are introduced based on the PQX distribution to predict the monthly counts of the earthquakes with magnitude 4 and above in Turkey. Empirical results show that the INARPQX(1) processes perform better than INARP(1), INARP(1), INARG(1), and INARNB(1) processes for the data used. As a future work of this study, we will try to extend the INARPQX(1) to multivariate case for joint modeling of the more than one region in predicting the monthly counts of earthquakes. We believe that the PQX distribution will increase its popularity find a wider application area in different sciences such medical, finance, and actuarial.
